# The role of bone cement for the development of intraoperative hypotension and hypoxia and its impact on mortality in hemiarthroplasty for femoral neck fractures

**DOI:** 10.1080/17453674.2020.1745510

**Published:** 2020-04-02

**Authors:** Fredrik Olsen, Mathias Hård Af Segerstad, Bengt Nellgård, Erik Houltz, Sven-Erik Ricksten

**Affiliations:** Department of Anesthesiology and Intensive Care Medicine, Institute of Clinical Sciences, Sahlgrenska Academy, University of Gothenburg and Sahlgrenska University Hospital, Gothenburg, Sweden

## Abstract

Background and purpose — The bone cement implantation syndrome characterized by hypotension and/or hypoxia is a well-known complication in cemented arthroplasty. We studied the incidence of hypotension and/or hypoxia in patients undergoing cemented or uncemented hemiarthroplasty for femoral neck fractures and evaluated whether bone cement was an independent risk factor for postoperative mortality.

Patients and methods — In this retrospective cohort study, 1,095 patients from 2 hospitals undergoing hemiarthroplasty with (n = 986) and without (n = 109) bone cementation were included. Pre-, intra-, and postoperative data were obtained from electronic medical records. Each patient was classified for grade of hypotension and hypoxia during and after prosthesis insertion according to Donaldson’s criteria (Grade 1, 2, 3). After adjustments for confounders, the hazard ratio (HR) for the use of bone cement on 1-year mortality was assessed.

Results — The incidence of hypoxia and/or hypotension was higher in the cemented (28%) compared with the uncemented group (17%) (p = 0.003). The incidence of severe hypotension/hypoxia (grade 2 or 3) was 6.9% in the cemented, but not observed in the uncemented group. The use of bone cement was an independent risk factor for 1-year mortality (HR 1.9, 95% CI 1.3–2.7), when adjusted for confounders.

Interpretation — The use of bone cement in hemiarthroplasty for femoral neck fractures increases the incidence of intraoperative hypoxia and/or hypotension and is an independent risk factor for postoperative 1-year mortality. Efforts should be made to identify patients at risk for BCIS and alternative strategies for the management of these patients should be considered.

The bone cement implantation syndrome (BCIS) is a well-recognized and potentially fatal complication of orthopedic surgery involving pressurized bone cement (Donaldson et al. 2009). The syndrome is mostly noted in cemented hemiarthroplasty after displaced femoral neck fractures, but is also found in total hip and knee replacement surgery (Byrick et al. 1986, Clark et al. 2001). This syndrome is characterized by hypoxia, systemic hypotension, pulmonary hypertension, arrhythmias, loss of consciousness, and cardiac arrest (Clark et al. 2001, Kotyra et al. 2010).

The pathophysiology of BCIS is unclear, but anaphylaxis, inflammatory, thermic and complement activation (Dahl et al. 1988) have all been implicated to induce BCIS (Donaldson et al. 2009). Studies employing invasive hemodynamic monitoring and perioperative ultrasound imaging have revealed subclinical pulmonary embolisms and hemodynamic changes, not detected in standard intra- and postoperative monitoring (Orsini et al. 1987, Bisignani et al. 2008, Kotyra et al. 2010).

Until recently, the incidence of BCIS in cemented hemiarthroplasty for hip fractures has been unknown, mainly because a consensual definition of the BCIS syndrome has been lacking. Donaldson et al. (2009) defined a severity classification of BCIS (Grade 1, 2, and 3). In a previous study on patients undergoing cemented hemiarthroplasty for hip fractures, we found that the incidence for all grades of BCIS was 28%, with a huge impact on early and late mortality (Olsen et al. 2014).

In this study, we evaluated the role of the cementation, per se, for the development of hypotension and hypoxia and its impact on mortality in patients undergoing hemiarthroplasty for femoral neck fractures. To enable this, a multitude of risk factors influencing mortality were collected in order to isolate the cementation effect. Our hypothesis was that the use of bone cement is an independent risk factor for postoperative mortality.

## Patients and methods

Patients undergoing hemiarthroplasty for femoral neck fracture in 2 hospitals in the south-west of Sweden, from January 2008 until July 2011, were included. The 1st cohort of patients was operated at Sahlgrenska University Hospital/Mölndal, which routinely performs cemented hemiarthroplasty in patients with femoral neck fracture. The second cohort was operated at Alingsås Regional Hospital, which routinely performs hemiarthroplasty without cement in patients with femoral neck fracture. The operative treatments were standard procedures at the respective hospitals. Patients were excluded when having a failed internal fixation or pathological fractures. When surgery was performed > 1 during the study period, only the initial operation was included.

From the review of the medical records, data were obtained retrospectively by 1 of the investigators (FO), for pre-existing comorbidity, medication, and biochemical markers. Besides sex, age, preadmission residence (assisted living), functional status (reduced mobility), ASA risk score, and type of anesthesia (regional or general), we collected data regarding preoperative cardiac history and presence of coexisting diseases, including liver disease, renal failure (defined as a documented history of renal failure and/or a serum creatinine >150 µmol/L), diabetes mellitus, previous stroke, peripheral vascular disease, hypertension, chronic obstructive pulmonary disease, cancer, dementia, arrhythmias, anemia, and current drug therapy. The selection of these variables was guided by a comprehensive review of the literature and subject-matter knowledge. Thus, in the present study we were able to retrieve from medical records 85% of previously described demographic factors and comorbidity associated with postoperative mortality (Maxwell et al. 2008, Juliebo et al. 2010, Olsen et al. 2014, Sheehan et al. 2016).

In all patients, anesthesia charts were reviewed for arterial oxygen saturation, mean arterial blood pressure, and heart rate. At the involved hospitals, these variables are recorded, routinely, immediately before induction of anesthesia and every 5th minute during the operation. In patients receiving cemented hemiarthroplasty, the cementation process is marked on the anesthesia chart. These variables were obtained on 4 occasions: (a) immediately prior to induction of anesthesia, (b) every 5th minute, for a period of 10–15 minutes before prosthesis insertion, (c) every 5th minute, for a period of at least 15 minutes after prosthesis insertion (with or without implantation of bone cement), and (d) on arrival at the post-anesthesia recovery unit. The lowest systolic blood pressure recorded within 15 minutes after prosthesis insertion was compared with pre-insertion values and used to score the severity of hypoxia/hypotension. Each patient was classified as having no hypotension/hypoxia (Grade 0) or Grade 1, 2, or 3 hypotension/hypoxia, according to the criteria of Donaldson et al., where Grade 1 was defined as moderate hypoxia (arterial oxygen saturation < 94%) or moderate hypotension (a decrease in systolic arterial pressure (SAP) > 20%), Grade 2 included severe hypoxia (arterial oxygen saturation < 88%) or severe hypotension (a decrease in SAP > 40%) or unexpected loss of consciousness, and finally Grade 3 was defined as a cardiovascular collapse requiring cardiopulmonary resuscitation

### Statistics

For descriptive purposes, categorical variables are presented with n (%), and continuous variables with mean (SD). In a univariable Cox proportional hazard regression analysis, we screened for important risk factors that were significantly associated with 1-year mortality (see Table 3). Unadjusted hazard ratios (HR) with 95% confidence interval (CI) were calculated for each risk factor. In addition, the following previously described risk factors (see above) for mortality were added: diabetes, stroke, and previous myocardial infarction. Thus, regarding the causality of the use of cement on 1-year mortality, the following 21 confounding variables were adjusted for in a multivariate Cox proportional hazard regression model; sex, age, assisted living, reduced mobility, ASA score, liver disease, renal failure, diabetes, stroke, arteriosclerosis, peripheral vascular disease, previous myocardial infarction, congestive heart failure, cancer, arrythmia, anemia (hemoglobin < 100g/L), use of diuretics, statins, antiplatelets drugs or ß-adrenergic blockers, and dementia. The adjusted hazard ratio for the variable bone cementation with CI is presented. The variable hypotension and/or hypoxia was considered a mediator variable and was not adjusted for in the Cox regression. None of the variables were considered colliders. The assumption of Cox proportional hazard was checked by using scaled Schoenfeld residuals and found satisfactory.

**Table 3.** Univariable unadjusted hazard ratios (HR) for 1-year mortality

Variables	HR (95% CI)	p-value
Cemented vs. uncemented	2.5 (1.5–4.2)	0.001
Male	1.5 (1.2–1.9)	< 0.001
Age at surgery (per 1 year)	1.07 (1.05–1.09)	< 0.001
Assisted living	2.7 (2.2–3.3)	< 0.001
Reduced mobility	1.7 (1.4–2.1)	< 0.001
ASA (1–2 vs. 3–4)	2.1 (1.7–2.7)	< 0.001
Regional anesthesia (spinal)	1.1 (0.8–1.5)	0.79
Medical history		
Liver disease	2.5 (1.1–5.7)	0.03
Renal failure	2.4 (1.5–3.8)	< 0.001
Diabetes	0.98 (0.69–1.4)	0.9
Stroke	1.3 (0.96–1.7)	0.1
Peripheral vascular disease	1.8 (1.0–3.2)	0.05
Arteriosclerosis	1.0 (0.91–3.2)	0.1
Hypertension	0.93 (0.74–1.2)	0.5
Angina pectoris	1.3 (0.92–1.7)	0.2
Previous myocardial infarction	1.3 (0.94–1.8)	0.1
Congestive heart failure	1.9 (1.4–2.6)	< 0.001
Chronic obstructive pulmonary		
disease	0.95 (0.67–1.4)	0.8
Cancer	1.7 (1.2–2.4)	0.002
Dementia	2.3 (1.8–2.8)	< 0.001
Arrhythmia	1.6 (1.2–2.0)	< 0.001
Medication		
ß-blockers	1.5 (1.2–1.8)	0.001
Diuretics	1.7 (1.5–2.2)	< 0.001
Antiplatelet drugs	1.5 (1.2–1.9)	0.001
Nitrates	1.3 (0.97–1.8)	0.08
Calcium antagonists	0.92 (0.68–1.2)	0.6
ACE inhibitors	1.0 (0.78–1.3)	0.9
Insulin	1.1 (0.68–1.7)	0.8
Warfarin	0.82 (0.49–1.4)	0.4
Statins	0.60 (0.40–0.88)	0.01
Preoperative		
hemoglobin < 100 g/L	1.8 (1.1–2.9)	0.04
serum creatinine > 150 µmol/L	2.3 (1.5–3.5)	< 0.001
Grade of hypotension/hypoxia		
(2–3 vs. 0–1)	4.1 (3.0–5.8)	< 0.001

Diabetes includes both types I and II. ASA;

ACE = angiotensin converting enzyme.

Unadjusted hazard ratios were calculated for each variable.

We also performed 2 sensitivity analyses to assess the robustness of our model and the role of residual confounding. In 1 sensitivity analysis, we performed a Cox regression including only the strongest covariates (n = 9) as found by us and others (see above), i.e., age, assisted living, ASA risk score, liver disease, renal failure, diabetes, stroke, myocardial infarction, and dementia. In another sensitivity analysis, we used the E-value methodology, as described by VanderWeele and Ding (2017), which estimates the minimum strength of association (risk ratio, 95% CI) that an unmeasured or uncontrolled confounding would need to have (on a risk ratio scale) with both the treatment and outcome to fully explain away an association between treatment and outcome. A large E-value indicates that considerable unmeasured confounding would be necessary to explain away an effect estimate. All tests were 2-tailed and conducted at 5% significance level. Statistical analysis was performed using SPSS version 25 (IBM Corp, Armonk, NY, USA).

### Ethics, data-sharing, funding, and potential conflicts of interest

The study was approved by the Gothenburg Regional Ethics Committee and written informed consent was waived by the Ethics Committee (IRB#887-16). Data may be shared on reasonable request. The study was supported by Swedish State Support for Clinical Research (LUA-ALF #75130) and the Gothenburg Medical Society. None of the authors report any conflict of interests.

## Results

1,159 patients were enrolled into the study. 64 patients were excluded from analysis due to registration errors (n = 30), lack of perioperative documentation (n = 3), and for indication other than acute fracture (n = 31). Thus, 109 patients were included in the uncemented group and 986 patients were included in the cemented group for analysis.

Baseline characteristics are given in Table 1. The cemented group was older and had a higher ASA classification and a higher incidence of dementia, compared with the uncemented group. The use of spinal anesthesia was more common in the uncemented group (96% vs. 86%). Preoperative medication with ß-adrenergic blockers, diuretics, and calcium antagonists was more frequent in the cemented group. Preoperative serum creatinine was higher in the cemented compared with the uncemented group.

**Table 1.** Baseline characteristics. Values are frequency (%) unless otherwise specified

	Uncemented	Cemented
Variable	(n = 109)	(n = 986)
Sex		
Female	80 (73)	704 (71)
Male	29 (27)	282 (29)
Age at surgery (SD)	82 (8)	85 (6)
Assisted living	48 (41)	570 (56)
Reduced mobility	38 (32)	422 (43)
ASA preoperative risk score:		
1	6 (5.6)	21 (2.1)
2	60 (56)	384 (39)
3	41 (38)	532 (54)
4	1 (0.9)	49 (5.0)
Spinal anesthesia	104 (96)	847 (86)
Medical history		
Liver disease	0 (0.0)	10 (1.0)
Renal failure	2 (1.8)	36 (3.7)
Diabetes	11 (10)	133 (14)
Stroke	16 (15)	183 (19)
Peripheral vascular disease	1 (0.9)	25 (2.5)
Arteriosclerosis	3 (2.8)	21 (2.1)
Hypertension	37 (34)	410 (42)
Angina pectoris	16 (15)	139 (14)
Previous myocardial infarction	11 (10)	111 (11)
Congestive heart failure	7 (6.4)	112 (11)
Chronic obstructive pulmonary		
disease	11 (10)	124 (12)
Cancer	11 (10)	66 (6.7)
Dementia	19 (17)	259 (26)
Arrhythmia	21 (19)	220 (22)
Medication		
β-adrenergic blocker	29 (27)	366 (37)
Diuretics	22 (20)	367 (37)
Antiplatelet drugs	41 (38)	422 (43)
Organic nitrates	14 (13)	139 (14)
Calcium antagonists	11 (10)	198 (20)
ACE inhibitors	19 (17)	235 (24)
Insulin	4 (3.7)	66 (6.7)
Warfarin	6 (5.5)	67 (6.8)
Statins	9 (8.3)	138 (14)
Preoperative hemoglobin (g/L) (SD)	127 (14)	125 (15)
Serum creatinine (µmol/L) (SD)	72 (25)	86 (41)

Renal failure is defined as serum creatinine > 150 µmol/L.

Diabetes includes both types I and II.

ACE = angiotensin converting enzyme.

Cumulative 1-year survival, adjusted for covariates, after hemiarthroplasty for femoral neck fracture with (red) or without (blue) the use of bone cement.

The incidence and grade of hypoxia and/or hypotension, according to Donaldson’s criteria, were higher and more pronounced, respectively, in the cemented group (p = 0.003) (Table 2). Thus, in patients with cemented hemiarthroplasty, 28% had symptoms of hypoxia/hypotension (Grade 1–3), compared with 17% in those with uncemented hemiarthroplasty. The incidence of severe hypotension/hypoxia, i.e. Grades 2 and 3, was 7% in the cemented group, while no patients in the uncemented group experienced severe hypotension and/or hypoxia.

**Table 2.** Incidence of intraoperative hypotension/hypoxia. Values are frequency (%)

	Uncemented	Cemented
Grade of hypotension/hypoxia	(n = 109)	(n = 986)
0	90 (83)	711 (72)
1	18 (17)	204 (21)
2	0 (0.0)	51 (5.2)
3	0 (0.0)	17 (1.7)

p = 0.003

Early postoperative mortality (< 48 hours) was 0% and 2% in the uncemented and cemented groups, respectively (p = 0.001). 30-day mortality was 3% in the uncemented and 9% in the cemented group (p = 0.03). One-year mortality was 13% in the uncemented group and 29% in the cemented, respectively (p < 0.001).

Univariable hazard ratios (HR) were calculated for a multitude of possible risk factors correlated to mortality (Table 3). We found that several factors indicating cardiovascular and respiratory comorbidity had high HRs (Table 3). Male sex, age, ASA preoperative risk score, assisted living, reduced mobility, liver disease, renal failure, peripheral vascular disease, congestive heart failure, cancer, dementia, and arrhythmia all showed high hazard ratios in the univariable analysis. Regarding pre-existing medication, the use of beta-blockers, diuretics, and anti-platelet drugs were all significantly associated with an increased HR. Conversely, the use of statins was associated with significantly reduced HR (0.6). When investigating laboratory data, lower levels of hemoglobin (< 100 g/L) and elevated serum creatinine levels (> 150 µmol/L) were associated with increased HR. The presence of intraoperative severe hypotension and/or hypoxia (Grade 2 and 3) and the use of bone cement were both univariably associated with higher mortality (p < 0.001, and p = 0.001, respectively)

In the Cox multivariate regression analysis, hypotension and/or hypoxia was not included as it was considered as a mediator variable. After adjustments for confounders (see above) in the multivariate Cox regression analysis, the use of bone cementation was associated with significantly increased HR for 1-year mortality (1.9, CI 1.3–2.7, Figure). In the 1st sensitivity analysis, including the most important confounders, the use of bone cementation was associated with significantly increased HR for 1-year mortality (1.8, CI 1.2–2.6). The calculated E-value was 3.1, i.e., a risk ratio of 3.1 (95% CI 1.9–4.8) for unmeasured confounding, both with outcome and with group, above the confounders already in the model, is needed to fully explain away our effect estimate of 1.9. Several models for variable selection were performed including clinically important risk factors, showing consistent hazard ratios and E values (Table 4, See Supplementary data).

**Figure:**
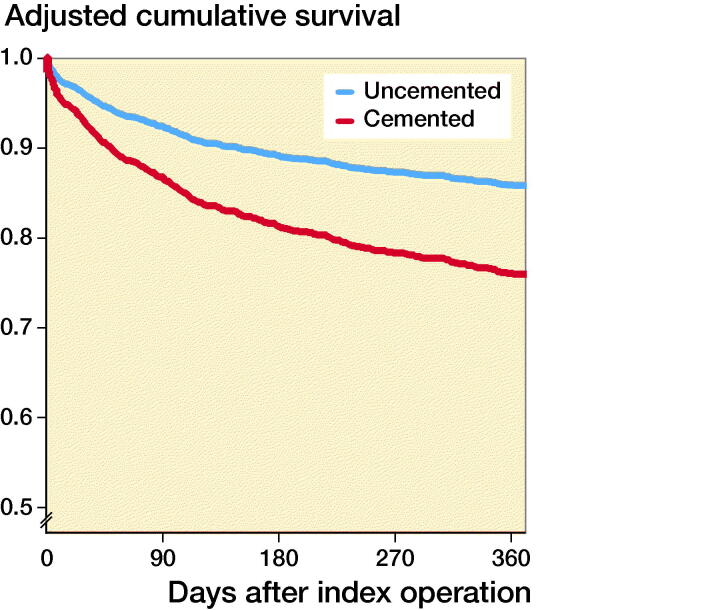


## Discussion

In the present investigation on patients undergoing cemented or uncemented hemiarthroplasty for femoral neck fractures, we used the classification system proposed by Donaldson et al, (2009), to evaluate the role of bone cementation for the development of intraoperative hemodynamic and pulmonary derangement and its impact on postoperative mortality. The main findings were that the incidence of hypoxia and/or hypotension was higher in the cemented compared with the uncemented group. In contrast, patients undergoing uncemented hemiarthroplasty, severe intraoperative hypotension, and/or hypoxia (Grades 2 or 3) were not observed. Furthermore, the use of bone cement was an independent risk factor for 1-year postoperative mortality.

To our knowledge, this is the first study demonstrating the link between the use of bone cement, per se, with intraoperative hemodynamic and/or pulmonary instability and increased postoperative mortality. It has been suggested that the BCIS is caused by an increase in intra-medullary pressure caused by prosthesis insertion and cementation, which will force medullary content into the circulation causing lung embolization (de Froidmont et al. 2014). This will, in turn, induce hypoxia, pulmonary vasoconstriction, and an increase in right ventricular (RV) afterload and eventually RV failure with systemic hypotension. Pulmonary embolization has been demonstrated in several studies by the use of transesophageal echocardiography in patients undergoing both uncemented and cemented total hip arthroplasty starting during reaming of the femur and acetabulum until the end of the procedure (Lafont et al. 1994). Pulmonary embolization has been shown to be particularly marked when placing the femoral component in a cementing procedure (Kotyra et al. 2010). However, it is not immediately evident that pulmonary embolization in cemented hip arthroplasty triggers intraoperative hemodynamic and/or pulmonary instability, as in those small studies episodes of lung embolization caused only minimal changes in pulmonary and systemic hemodynamics with few adverse clinical events (Kotyra et al. 2010). In the present study, however, including more than 1,000 patients undergoing hemiarthroplasty for femoral neck fractures, the use of bone cement independently predicted 30-day mortality.

Our data support the results from a recent study by White et al. (2016), showing that a fall in in blood pressure, intraoperatively, is significantly associated with a higher 5-day and 30-day mortality in patients undergoing hip fracture surgery. These findings are in line with recent studies showing that intraoperative hypotension is associated with increased postoperative morbidity and 30-day mortality in patients undergoing non-cardiac surgery (Walsh et al. 2013, Monk et al. 2015). Thus, the combination of a reduced perfusion pressure and arterial oxygen content, intraoperatively, seems to have an important role in the development of postoperative organ injury and mortality.

To investigate changes in systemic and pulmonary hemodynamics, Kotyra et al. (2010) inserted pulmonary artery catheters in anesthetized patients operated on with cemented hemi-arthroplasty after an acute hip fracture. A clear temporal association with cementation and a distinct 45% increase in pulmonary vascular resistance (PVR) was found. The increase in PVR was clinically apparent only in 1/15 patients in that study, suggesting that embolization from the medullary canal is present in all cases and that the patients’ capability of handling a sudden increase in PVR determines the degree of hemodynamic instability, expressed clinically, heavily influenced by comorbidity and age.

An analysis of the degree of hypotension and/or hypoxia in patients undergoing uncemented hemiarthroplasty for femoral fractures applying the Donaldson criteria has, to our knowledge, not previously been published. Severe hemodynamic and/or pulmonary instability was not seen in any of the 109 patients undergoing uncemented hemiarthroplasty, despite the fact that this group consisted of patients with a high ASA score (ASA 3 ≈ 40%) and a high incidence of chronic obstructive pulmonary disease and congestive heart failure, all being associated with severe intraoperative hypotension and/or hypoxia in cemented hemiarthroplasty (Olsen et al. 2014). This strongly suggests that the bone cement itself is required to induce severe intraoperative hypotension and/or hypoxia with negative consequences on postoperative survival.

We found that early (< 48 hours) postoperative mortality was 2% in the cemented group, a result somewhat higher than in previous studies showing an early mortality of 0.8–1.3% after cemented hemiarthroplasty. In a previous study, we identified risk factors for the development of BCIS and found that BCIS Grade 2 or 3 was associated with a 16-fold increase in 30-day mortality (Olsen et al. 2014). Thus, patients at risk for developing intraoperative hypotension and/or hypoxia are more likely to succumb early, if cement is used. Costain et al. (2011) could demonstrate that mortality from cemented hemiarthroplasty occurs mainly in the perioperative period. Thereafter the survival did not differ from the group not receiving bone cement. However, to perform preventive measures in patients undergoing cemented hemiarthroplasty to lower the risk of developing BCIS and to decrease early mortality, efforts should be made to identify patients at risk for BCIS. Furthermore, one should question the use of cemented hemiarthroplasty in elderly, male patients with a high ASA score and compromised cardiac and renal function, despite the fact that the alternative, cementless hemiarthroplasty, is associated with an increased rate of postoperative surgical complications and a worse biomechanical performance (Khan et al. 2002, Parker et al. 2010, Li et al. 2013, Ekman et al. 2019). The risk of postoperative morbidity and mortality, as well as patient satisfaction and the surgical result, all have to be evaluated when choosing which procedure to pursue.

Our results are at variance with prospective randomized trials on patients undergoing hemiarthroplasty for femoral neck fracture showing no difference in 1-year mortality between cemented and uncemented hemiprosthesis (Parker et al. 2010, Li et al. 2013, Talsnes et al. 2013). Although we made efforts to adjust for differences in baseline demographic characteristics, preoperative risk score, type of anesthesia, comorbidity, medication, and renal function, we cannot rule out the possibility that there might be residual unknown confounding variables not recorded by us that to some extent could explain the difference in one-year mortality between the two groups.

Our study is limited by its retrospective design. Therefore, we were bound by the quality of data presented in the medical records from both hospitals. In order to avoid inter-observer variability, the extraction of data in both hospitals was performed by 1 of the investigators (FO). The study groups varied in size by a factor of 10. A post-hoc power analysis revealed, however, that the smaller study group having uncemented hemiarthroplasty was adequate to reach our desired endpoints. Furthermore, we have no information on the cause of death. In addition, although we have adjusted for several known confounders, confounding may persist. Several potential unmeasured confounders are not accounted for in our model; these include, but are not limited to, surgeon and anesthesiologist seniority, time of admission, BMI, and smoking amongst others. In addition, surgical delay, an important systemic factor for mortality (Sheehan et al. 2016), was not available to us at the time of data collection and should also be regarded as an unmeasured confounder. On the other hand, we believe that the evidence of association between bone cementation and 1-year mortality seems reasonably robust to residual confounding, as substantial unmeasured confounding would be necessary to negate the observed association. We are unaware of any potential confounder with such a strong risk (HR ≥ 3), above those already in the model, that is not already included in our model. The strength of the study is that we have analyzed individual anesthesia charts of more than 1,000 patients undergoing hemiarthroplasty for femoral neck fractures with or without bone cementation, identifying patients with more or less severe hemodynamic and pulmonary instability at the time around prosthesis insertion.

In conclusion, in this retrospective study, on patients undergoing hemiarthroplasty for femoral neck fractures, we found that the use of bone cement was an independent risk factor for 1-year postoperative mortality. Cemented hemiarthroplasty is the preferred technique for displaced neck of femur fracture from an orthopedic surgeon’s perspective. Thus, with our results at hand, it is pivotal for a successful outcome that these patients are discussed preoperatively in an interdisciplinary forum focusing on risk identification and intraoperative surgical strategy, to mitigate the risk for intraoperative hypotension and/or hypoxia and mortality.

## Supplementary Material

Supplemental Material

## Supplementary data

Table 4 is available as supplementary data in the online version of this article, http://dx.doi.org/10.1080/17453674.2020.1745510
