# Supplementary material for: The role of bone cement for the development of intraoperative hypotension and hypoxia and its impact on mortality in hemiarthroplasty for femoral neck fractures
Source: Acta Orthop. 2020 Apr 2;91(3):293–8. doi: 10.1080/17453674.2020.1745510 (PMC8023921; doi:10.1080/17453674.2020.1745510)
Supplement: Supplemental Material [file IORT_A_1745510_SM4407.pdf]

Supplementary data

Table 4. Cox regressions on combinations of clinically important risk factors for outcome

Variables	number	HR (95% CI)	p-value	E-value (95% CI)
Living, mobility, sex, age, ASA, ß-blocker, diuretics, antiplatelet drugs, statin, liver disease, diabetes, stroke, peripheral vascular disease, arteriosclerosis, previous myocardial infarction, congestive heart failure, cancer, dementia, arrythmia, renal failure, anemia	21	1.9 (1.3–2.7)	0.001	3.1 (1.8–NA)
Living, sex, ASA, age, mobility	5	1.8 (1.2–2.6)	0.002	2.9 (1.8–NA)
Age, ASA, living, renal failure, liver disease, dementia, previous myocardial infarction, diabetes, stroke	9	1.8 (1.2–2.6)	0.002	3.0 (1.8–NA)
Sex, ASA, age, living, liver disease, renal failure, dementia, previous myocardial infarction, diabetes, stroke, mobility, peripheral vascular disease, arteriosclerosis, congestive heart failure, cancer, arrythmia, diuretics, statins	18	1.8 (1.3–2.7)	0.001	3.1 (1.8–NA)
Sex, ASA, age, living, liver disease, renal failure, dementia, mobility, peripheral vascular disease, arteriosclerosis, congestive heart failure, cancer, arrythmia, diuretics, statins	15	1.8 (1.3–2.7)	0.001	3.1 (1.9–NA)
ASA, age, living, renal failure, liver disease, dementia	6	1.7 (1.2–2.5)	0.004	2.8 (1.7–NA)

Irrespective of the number and combinations of these risk factors, the HR for bone cementation ranges from 1.7 to 1.9 with p-values 0.004 to 0.001
